# What Makes Tourist Experiences Interesting

**DOI:** 10.3389/fpsyg.2019.01603

**Published:** 2019-08-07

**Authors:** Svein Larsen, Katharina Wolff, Rouven Doran, Torvald Øgaard

**Affiliations:** ^1^Department of Psychosocial Science, Faculty of Psychology, University of Bergen, Bergen, Norway; ^2^Norwegian School of Hotel Management, Faculty of Social Sciences, University of Stavanger, Stavanger, Norway

**Keywords:** interesting tourist experience, novelty, familiarity, tourist roles, interaction hypothesis of interest

## Abstract

Traditional tourist role theory implies that tourists are *either* novelty seekers *or* familiarity seekers, while the interaction-hypothesis-of-inherent-interest predicts that *interestingness* is maximal when novel *and* familiar elements simultaneously are present in the experience. This paper tests these conflicting theoretical perspectives in three large surveys. In Study 1 (*N* = 1,029), both novelty and familiarity seeking tourists were asked about how interesting it would be for them to meet tourists from their home country (familiar) or from a foreign country (unfamiliar), either at home (familiar) or abroad (unfamiliar). Study 2 (*N* = 760) asked tourists to indicate the interestingness of well-known (familiar) and unknown (unfamiliar) sights at home (familiar) and abroad (unfamiliar) in familiarity seekers and novelty seekers alike. Study 3 (*N* = 1,526) was a field experiment were tourists rated interestingness of familiar and unfamiliar attractions in familiar and unfamiliar surroundings for either themselves or for other tourists. Results show that perceived interestingness of tourist experiences depends on a combination of familiarity and novelty, for both familiarity seekers and novelty seekers. These results therefore are supportive of the interaction-hypothesis-of-inherent-interest; seemingly cognitive factors are better predictors of interestingness of tourist experiences than personality is.

## Introduction

Understanding the tourist experience has been a major scholarly task for as long as tourism research has existed. Various social sciences, such as, for example, sociology (e.g., Cohen, 1972, 1979; Crompton, 1979; MacCannell, 1999; Uriely, 2005), social anthropology and ethnology (e.g., Graburn, 1983; Yiannakis and Gibson, 1992; MacCannell, 1999; O’Dell, 2007; Selstad, 2007), marketing and economics, (e.g., Andersson, 2007; Mossberg, 2007), and psychology (e.g., Mannell and Iso-Ahola, 1987; Pearce and Stringer, 1991; Vittersø et al., 2000, 2001; Larsen, 2007) have approached the tourist experience under a plethora of headlines, based on different types of data (or, sometimes with no systematic data), with a number of aims and contents, and with rampant methodological flexibility.

But it is still safe to say that tourist experiences are under researched (Yiannakis and Gibson, 1992; Larsen et al., 2007, 2017; Pearce and Packer, 2012) and only rudimentarily understood. This may partly be because tourism studies are inherently multi-disciplinary. It may well be that the disciplines do not always understand each other (Pearce and Packer, 2012). Also, disciplines focus on different aspects of “experiences,” levels of analysis differ, methodologies differ, technical terms differ and may imply different meanings, and the social sciences vary concerning what kind of data and research designs are acceptable (Larsen et al., 2017). The way toward a unified theory of tourist experiences seems to be hampered with ontological as well as epistemological problems, both between and within disciplines.

The present paper therefore sets out to test two opposing perspectives on the tourist experience derived from sociology (Cohen, 1972) and cognitive psychology (Teigen, 1985a,b,c, 1987), with the aim of comparing these perspectives in terms of their predictions. On the one hand, the sociological model (Cohen, 1972) predicts that tourists are different from each other in terms of their tourist roles; some tourists are novelty seekers and some are familiarity seekers. On the other hand, the cognitive psychological model (Teigen, 1985a,b,c, 1987) predicts that people, no matter their tourist role orientation, are inherently similar in terms of what constitutes an “interesting tourist experience.” This cognitive model challenges the dichotomy of novelty and familiarity in claiming that general psychological processes underlie the experience of interestingness, not individual differences in tourist role orientations or in tourists’ preferences. Knowledge about which perspective makes the best predictions is inherently important for theory-development within psychology. In addition, if the tourist industry has sound knowledge of what the generic aspects of interestingness are, then customization of tourist products and services may be improved (Larsen, 2007).

### Literature Review

Traditional tourist role theory (e.g., Cohen, 1972, 1979; Snepenger, 1987; Yiannakis and Gibson, 1992; Mo et al., 1993) maintains that familiarity and novelty are opposites on a preference continuum. According to this model, tourists are *either* predominantly novelty seekers *or* predominantly familiarity seekers. Cohen (1972) emphasizes that *tourists* can be classified according to their degree of institutionalization. The “drifter,” who is characterized by his/her experimental mode of traveling which highlights his/her seek for novelty in relative strange environments, is the most independent of all the tourists in Cohen’s taxonomy, while the least novelty seeking tourist in this scheme is the “institutionalized mass tourist.”

In another seminal paper, Cohen (1979) once more proposes a descriptive scheme where five types of tourist groups are suggested. Such groups represent a number of modes of experiences which allocate individuals in segments of tourists varying from those who are mere recreation seeking to those who search for an existential meaning based on a hypothesized “center” which in one way or another resides in peoples’ minds. Snepenger (1987) and Mo et al. (1993) found some support for Cohen’s (1972) model, while Yiannakis and Gibson (1992) also found empirical evidence for the four roles of the Cohen (1972) scheme, in addition to several other tourist roles. Lepp and Gibson (2003) stated that tourists can be classified according to the degree of novelty and familiarity sought, thus highlighting that novelty seeking constitutes a motive in itself. In this line of thinking, the motive of novelty seeking represents the opposite of the familiarity seeking motive. Similar perspectives can be found in many publications within the literature on tourist roles and tourist motivation (e.g., Crompton, 1979; Gilbert, 1991; Yiannakis and Gibson, 1992; Elsrud, 2001; Lepp and Gibson, 2003).

A more recent study Hwang and Hyun (2016) found that luxury cruise passengers’ perception of cruise lines’ innovativeness (which can be seen as a proxy for novelty) is an important factor influencing various aspects of cruise travelers’ experience in the luxury market. Li et al. (2015) found that sensation seeking was a personality characteristic impacting tourist roles in as much as they asserted that sensation seekers would be more inclined to become independent tourists. These results imply that from the tourist role perspective, people are inherently different from each other in systematic ways that allows for the segmentation of customers according to a psychographic scheme (Snepenger, 1987). The tourist role perspective therefore predicts that tourists are either inclined to be novelty (sensation) seekers or familiarity seekers (Mo et al., 1993).

The interaction-hypothesis-of-interest however states that inherent interestingness of a given situation will be maximal for *everyone* when novel *and* familiar elements are present at the same time (Teigen, 1987). In other words, this theory predicts that no matter the personality of the tourist, interestingness is a function of interpretations of the stimulus situation, in our case the tourist destination, the tourist attraction or more generally the tourists’ on-line experience. In a series of experiments, Teigen addressed *informativeness* of verbal information (Teigen, 1985a), *preferences* for news as a function of familiarity (Teigen, 1985b), sources of *interest* in verbal information (Teigen, 1985c), and the *interaction of novelty and familiarity* for intrinsic interest (Teigen, 1987). These experimental studies jointly demonstrated that inherent interest is a function of the interplay of novelty and familiarity. For example, subjects expressed more interest for *news* about familiar themes than unfamiliar themes, and they preferred to learn *news* about familiar countries more than about unfamiliar countries. In addition, in the third experiment reported by Teigen (1987), the focus was on a social encounter; that is, meeting a (familiar/non-familiar) tourist on a destination, which varied on the familiarity/non-familiarity dimension. Results indicated that the less familiar the imagined destination was, the stronger the subjects preferred meeting familiar others. In a similar vein of thinking, Yiannakis and Gibson (1992) hinted that “the location of a particular tourist role… indicates the optimal balance of stimulation-tranquillity, familiarity-strangeness and structure-independence” (p. 299), but their data were used to locate individuals to various tourist roles in a multidimensional space. It was in other words the tourists who were allocated to various roles, not the generic aspects of the experience that was studied.

Surprisingly, Teigen’s (1985a,b,c, 1987) studies have not had much impact on the academic tourism literature (with the exception of Noone et al., 2009). This may partly be due to the fact that Teigen’s work was published in generic psychology journals. Such journals are seemingly not often consulted by tourism scholars, maybe because these journals are considered to be too technical, too limited in scope, too often based on experimental data (which some tourism scholars even judge to be of little value), and too generic and thus of limited relevance for the interdisciplinary studies of tourism and tourists. But, as Pearce and Packer (2012) underline “…the breadth and intense scrutiny of human behaviour and experience undertaken within psychology” (p. 386) represents a vast resource and a challenge for tourism scholars, a standpoint which is in line with the assumptions underlying the current study.

### Research Aims

**Figure 1:**
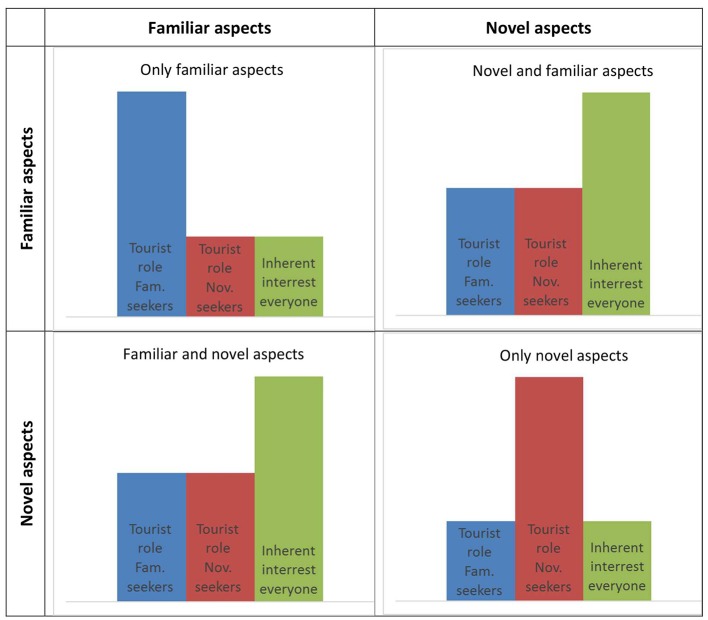
Predictions of interestingness by tourist role theory and interaction hypotheses.

The present research represents an attempt at testing the predictions of tourist role theory against the predictions of the interaction hypothesis of inherent interestingness. While tourist role theory predicts that people are inherently different from each other in what makes experiences interesting for them, the interaction hypothesis predicts that interestingness is a function of aspects of the experience, no matter who the individual is. Figure 1 shows the predictions made by these two theoretical perspectives.

As can be seen from Figure 1, predictions are opposite in these two perspectives. Tourist role theory predicts that situations containing a combination of high and low familiarity will be least interesting for both familiarity and novelty seekers, while the interaction perspective predicts that it is exactly these two conditions that will be the most interesting for all tourists. Consequently, it seems reasonable to put these two perspectives to the test: which makes the best predictions?

Three studies were undertaken. The studies were designed so that tourist role theory and inherent interest hypothesis would predict differential outcomes. Study 1 tested whether novelty seekers prefer to meet foreigners abroad (maximum novelty) while familiarity seekers prefer to meet compatriots at home (maximum familiarity), or whether *all* tourists prefer to meet compatriots abroad and foreigners at home (combination of novel and familiar stimuli). Study 2 validated the concept of interestingness. In addition, Study 2 planned to replicate the finding in Study 1 concerning preferences of tourists with various tourist role orientations. In Study 3, an attempt was made at removing tourists’ self-perception as being less institutionalized and more novelty seeking than other tourists (Prebensen et al., 2003; Doran et al., 2018) from the responses. Therefore it was hypothesized that when judging what is interesting for *other* tourists, tourists would judge a combination of novelty and familiarity of the experience to be most interesting and attractive.

## Study 1: Interestingness of Social Interaction

In accordance with the predictions of the interaction hypothesis of inherent interest, in Study 1, it was hypothesized that tourists would find it more interesting to meet a compatriot in an unknown place than in their home country. It was also expected that meeting a foreign tourist would be more interesting in a more familiar setting. We also tested whether tourists high on the novelty seeking motive differed from those low in this motive in terms of what they judged interesting in social encounters, which is the prediction of tourist role theory.

### Materials and Methods

Following the procedures indicated by Larsen et al. (2011), tourists were approached in “low threshold” places; that is, spaces that many tourists would “want to visit… and that none would be excluded for resource reasons, e.g., disabilities, high prices etc.” (p. 695), such as, for example, Mount Fløyen, the Tourist Information Office and the Fish Market in Bergen. Potential respondents were asked if they were on vacation, and if so, if they would be willing to fill in a questionnaire concerning “various aspects of being a tourist.” The questionnaire was two pages long, and it took some 5 min to fill it in. Standard background questions, such as age, gender, and nationality, were asked, in addition to *focus questions* where novelty and familiarity were manipulated (high and low familiarity of place and of social interaction). No monetary or other compensation was given for participation.

### Questionnaire

*Inherent interest* was measured by asking participants how interesting it would be for them to meet a tourist from Norway in “your home country,” Norway, Spain, Australia, and China, all measured on 7-point scales anchored by “Not interesting” (1) and “Very interesting” (7). To distinguish between *familiarity* and *novelty seekers*, three items addressing preference for “unorganized” and “organized” trips were used in accordance with the assumptions of Cohen’s tourist role scheme (Cohen, 1972, 1979). The items had the following form; “When I travel to…” (1) “…an exotic destination for the first time I prefer,” (2) “…to an exotic destination I have visited before I prefer,” and (3) “…to a destination I know well from before I prefer.” The preferences were indicated on 7-point scales anchored by “Unorganized individual trips” (1) and “Organized group trips” (7). The three items were treated as a scale (*α* = 0.78), and the quartile of the respondents scoring lowest on the scale were categorized as novelty seekers (*n* = 289), while the respondents with scores on the upper quartile (*n* = 271) were grouped as familiarity seekers. The remaining respondents (*n* = 469) scored in the mid-category (neither novelty nor familiarity seekers) and were thus excluded from the analyses concerning differences between novelty and familiarity seekers.

### Participants

Of some 1,200 approached tourists, 1,029 agreed to fill in the questionnaire. The respondents represented 52 nations, 49.1% were female and 49.1% male (1.8% did not answer the gender item). In addition to participants from countries investigated by questionnaire items concerning the target issues, respondents from countries with more than 40 respondents (i.e., USA, Germany, the United Kingdom, the Netherlands) were also included in the data analysis.

### Results

Table 1 shows how *interesting* tourists from Scandinavia (i.e., Norway, Sweden, and Denmark), Spain, Australia, China, USA, Germany, the United Kingdom, and the Netherlands would find it *to meet tourists from their home country* in their home country, in Norway, in Spain, in Australia, and in China. As can be seen from Table 1, tourists tend to judge meeting compatriots more interesting the further away (culturally and geographically) they are from their home country. Norwegian tourists, for example, judge meeting a Norwegian (high familiarity) to be significantly more interesting in China or Australia (high novelty) than in Norway (high familiarity) and Spain (moderate familiarity). Australian respondents indicate that it would be more interesting to meet Australians (high familiarity) abroad (high novelty) than at home (high familiarity). Similarly, Chinese tourists find Chinese tourists (high familiarity) more interesting abroad (high novelty) than at home (high familiarity). And the same is generally true for all the groups; the least interesting place to meet a compatriot is at home, the most interesting place to meet a person from ones’ home country is in a remote place, no matter what the home country of the individual tourist may be. This finding is stable over all nationalities and indicates that for tourists, familiarity of the place does not work well with familiarity of the social interaction, and vice versa, that high novelty of the place does not work well in harmony with high novelty of the social encounter.

**Table 1:** *Interestingness*^a^ of meeting a tourist *from one’s home country* in various countries [scale: 1 (not interesting) – 7 (very interesting), mean scores, ±SD].

Meet a tourist from your home country in…	…home country	…Norway	…Spain	…Australia	…China
Scandinavian^b^ tourists (*n* = 57)		3.14 ± 1.84	3.48 ± 1.72	4.32 ± 1.95	4.25 ± 2.00
Spanish tourists (*n* = 21)		4.16 ± 2.39	3.47 ± 2.45	4.61 ± 2.25	3.61 ± 1.95
Australian tourists (*n* = 41)		3.78 ± 1.90	3.78 ± 1.78	2.32 ± 1.49	3.51 ± 1.93
Chinese tourists (*n* = 23)		4.61 ± 1.92	4.30 ± 1.84	4.39 ± 1.80	2.70 ± 1.77
Tourists from USA (*n* = 138)	2.74 ± 1.87	4.21 ± 1.86	3.94 ± 1.94	4.03 ± 1.93	4.18 ± 1.95
German tourists (*n* = 136)	2.52 ± 1.92	3.18 ± 2.05	2.72 ± 1.97	3.18 ± 2.06	3.21 ± 2.13
UK tourists (*n* = 118)	2.94 ± 1.80	3.85 ± 1.88	3.47 ± 1.62	3.65 ± 1.86	3.75 ± 1.90
NL tourists (*n* = 72)	2.17 ± 1.46	3.12 ± 1.86	2.51 ± 1.37	2.98 ± 1.74	2.94 ± 1.87

*Dark fields indicate minimal interest (only familiar aspects) predicted by inherent interest hypothesis*.

a *All max-min differences within rows significant at p < 0.05 level, paired sample t-test*.

b *Tourists from Denmark, Norway, and Sweden*.

Table 2 shows that when the question was framed as meeting a Chinese tourist in various destinations (Norway, Spain, Australia, and China), the pattern is *exactly opposite* for all groups of respondents. Norwegians tend to judge meeting Chinese (low familiarity) in Norway (high familiarity) the most interesting. The same pattern emerges in the tourists from all other countries as well; Chinese tourists (high novelty) are thought of as being most interesting to meet in the tourists’ own home countries (high familiarity), but less interesting in China. In other words, the more remote the place in terms of distance or culture, the more interesting it will be to meet someone “more familiar,” and the more familiar the place is in terms of culture and distance, the less interesting it will be to meet familiar other tourists. Tourists in general it seems, prefer to meet compatriots and not local people when they travel to foreign countries that are new to them.

**Table 2:** *Interestingness*^a^ of meeting a tourist *from China* in various countries [scale: 1 (not interesting) – 7 (very interesting), mean scores, ±SD].

Meet a tourist from your home country in…	…home country	…Norway	…Spain	…Australia	…China
Scandinavian^b^ tourists (*n* = 57)		4.45 ± 1.92	3.05 ± 1.86	3.10 ± 1.98	3.05 ± 2.11
Spanish tourists (*n* = 21)		4.05 ± 1.82	4.29 ± 2.43	4.33 ± 1.71	3.24 ± 2.32
Australian tourists (*n* = 41)		3.78 ± 2.07	3.62 ± 1.98	3.81 ± 2.08	3.15 ± 2.06
Chinese tourists (*n* = 23)		4.21 ± 2.02	4.04 ± 2.08	4.08 ± 2.07	3.39 ± 2.06
Tourists from USA (*n* = 138)	4.20 ± 2.01	4.27 ± 2.00	3.95 ± 1.95	3.98 ± 2.00	3.41 ± 2.10
German tourists (*n* = 119–121)	3.55 ± 2.15	2.83 ± 1.94	2.68 ± 1.89	2.77 ± 1.90	2.84 ± 2.09
UK tourists (*n* = 108–110)	4.03 ± 1.98	3.41 ± 1.93	3.06 ± 1.84	3.40 ± 1.98	3.41 ± 2.10
NL tourists (*n* = 72)	2.61 ± 1.70	2.45 ± 1.79	2.04 ± 1.46	2.29 ± 1.67	2.47 ± 2.03

*Dark fields indicate maximal interest (familiar and novel aspects) predicted by inherent interest hypothesis*.

a *All max-min differences within rows significant at p < 0.05 level, paired sample t-test*.

b *Tourists from Denmark, Norway, and Sweden*.

The second issue, whether familiarity seekers and novelty seekers differ from each other in terms of their preference for novelty and familiarity of social encounters was examined by using the top and bottom quartiles in the distribution of the scale measuring preferences for novelty and familiarity.

**Figure 2:**
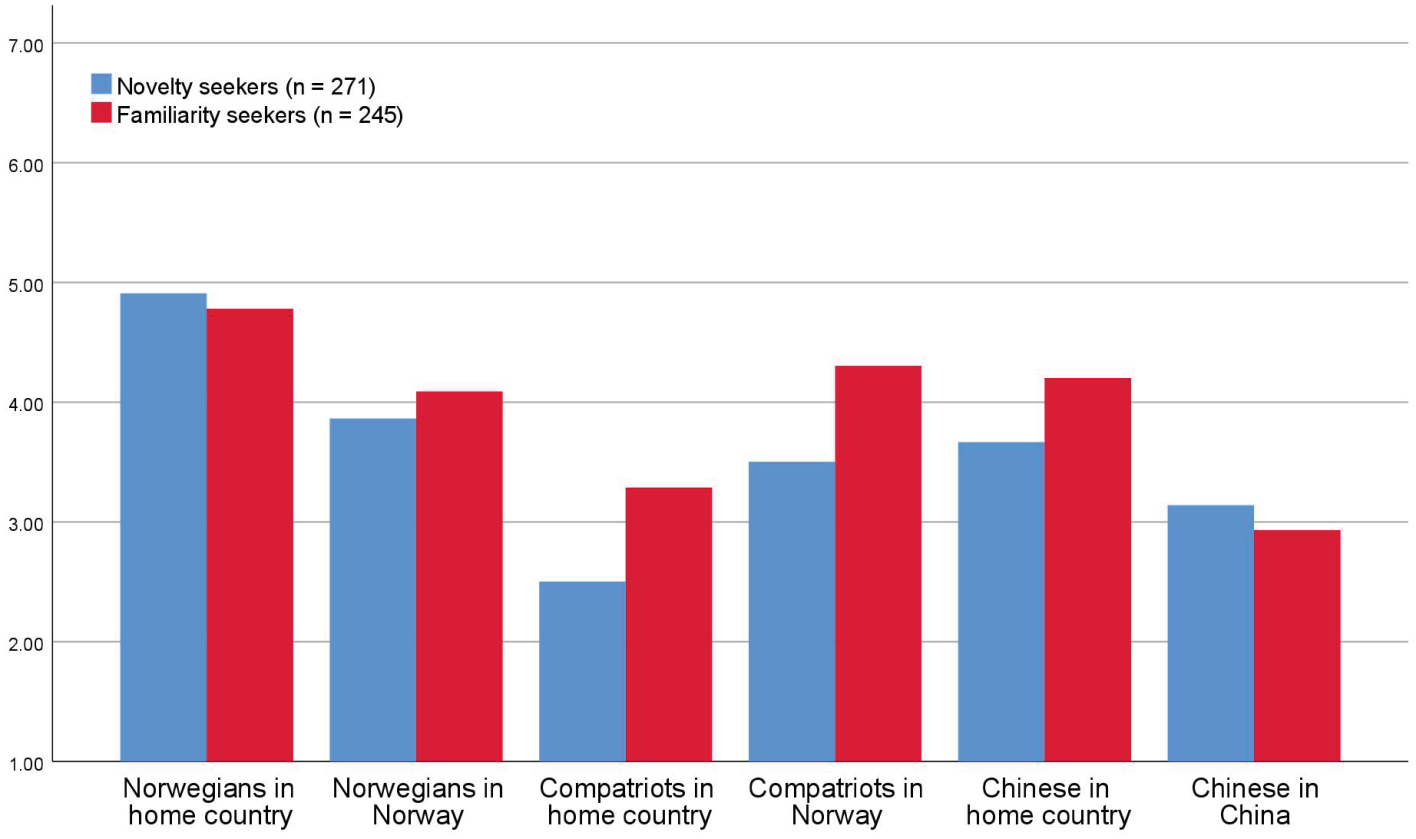
Interestingness of social encounters with locals and compatriots in novelty and familiarity seeking tourists [scale 1 (low interestingness) – 7 (high interestingness)].

Figure 2 exhibits an extrapolation of some of the very complex data concerning the mixture of familiarity and novelty of social encounters (Chinese and Norwegian respondents are for logical reasons removed from Figure 2). As can be seen, there are no differences in the preference *structure* concerning social encounters – both familiarity seekers and novelty seekers find it significantly more interesting to meet Norwegians (unfamiliar) at home (in a familiar place) than in Norway (unfamiliar place). At the same time, familiarity seekers and novelty seekers both find it more interesting to meet compatriots (familiar) in Norway (unfamiliar) than at home (familiar). The same holds true for meeting Chinese tourists; both familiarity seekers and novelty seekers judge meeting Chinese (unfamiliar) at home (familiar) more interesting than meeting Chinese (unfamiliar) in China (unfamiliar). It seems like *all* tourists, those who classify themselves as novelty seekers and those who are inclined to perceive themselves as familiarity seekers alike show the same structure of preferences for social encounters in familiar and unfamiliar settings.

### Discussion

Results from Study 1 give support to the interaction hypothesis of inherent interestingness for social encounters during tourist trips. Tourists seemingly prefer to meet compatriots abroad and foreigners at home. This appears to be true for both familiarity seekers and novelty seekers alike and for tourists from all countries. Since it was somewhat surprising that familiarity seekers and novelty seekers showed the same preference structure, Study 2 focused on whether novelty seekers and familiarity seekers demonstrate similar preference structures in other types of tourist experiences than social encounters. This issue is consequently addressed below.

## Study 2: Interestingness of Attractions in Familiarity and Novelty Seekers

Study 2 follows up the intriguing finding that novelty seekers and familiarity seekers report the same structure concerning interestingness reported in Study 1. Three measures of interestingness were used; “willingness to pay,” “attractiveness,” and “interestingness” with reference to four different conditions in a within subjects design; (1) unknown sights in a known place (home), (2) known sights in a known place (home), (3) unknown sights in a unknown place (away from home), and (4) known sights in an unknown place (away from home). Interestingness should correlate moderately highly with tourists’ willingness to pay for the experience and with their judgment of the attractions’ attractiveness. Based on the results from Study 1, it was hypothesized that tourists will judge the interestingness of tourist attractions to be the highest for known, i.e., familiar attractions in unknown destinations and for less known attractions in known settings, and lowest for known attractions in known destinations and unfamiliar attractions in unfamiliar places. Based on the results from Study 1, it was further expected that familiarity seekers and novelty seekers would demonstrate the same preference structures.

### Materials and Methods

Potential respondents were approached in “low threshold” places and asked if they were on vacation. If the potential participant answered this question in the affirmative, they were asked if they would be willing to fill in a questionnaire concerning various “aspects of being a tourist.”

### Questionnaire

The questionnaire was four pages long, and it took some 10 min to fill it in. Standard background questions, such as age, gender, and nationality, were asked. Tourist role orientation was measured using the 16-item version (Jiang et al., 2000) of the International Tourist Role Scale (ITR; Mo et al., 1993), a scale developed in order to empirically asses Cohen’s (1972) tourist role typology. In the present context, only the subscale measuring preference for familiarity when choosing a travel destination was used. Four scenarios describing novel and familiar aspects of places and sights were constructed. Respondents indicated interestingness (attractiveness/willingness to pay) for each of the scenarios (“hidden treasures in your hometown,” “famous landmarks in your hometown,” “hidden treasures in a town you visit for the first time,” and “famous landmarks in a town you visit for the first time”). All items were on 7-point scales anchored by “Not interesting” (or “attractive”/“no willingness to pay”) and “Very interesting” (“attractive”/“willing to pay”). No monetary or other compensations were given for participation.

### Participants

Of some 820 approached tourists, 762 agreed to fill in the questionnaire. The respondents represented 57 nations, 52.8% were female and 47.1% male (1 person did not answer the gender item). Mean age was 41 years (SD = 17.3).

### Results

Interestingness correlated highly with both attractiveness and willingness to pay (*r* ranges between 0.59 and 0.82) in all the four scenarios. Thus, it was decided that *interestingness* could be operationalized as a scale consisting of the three items measuring interestingness, attractiveness, and willingness to pay. This scale yielded Chronbach’s *α* for Scenario 1 (familiar place novel sight) = 0.86, Chronbach’s *α* for Scenario 2 (familiar place/familiar sight) = 0.87, Chronbach’s *α* for Scenario 3 (novel place/novel sight) = 0.90, and Chronbach’s *α* for Scenario 4 (novel place/familiar sight) = 0.91. This allows for construction of four interestingness scores with reference to the four scenarios.

In line with predictions from the interaction hypothesis, results indicated that tourists, when thinking about their hometown, in general report that they would find it more interesting to see unfamiliar (novel) sights than familiar sights (home: mean_(familiar/familiar)_ = 3.11, mean_(novel/familiar)_ = 3.38, *t* = 6.78, *p* < 0.001). At the same time, and contrary to the predictions of the interaction hypothesis of inherent interest, tourists reported that in unfamiliar (novel) places, they would find unfamiliar sights more interesting than well-known attractions in these places (away from home: mean_(novel/novel)_ = 4.85, mean_(familiar/novel)_ = 3.75, *t* = 3.17, *p* < 0.005). This structure of responses fits with the predictions made in traditional tourist role theory *for novelty seekers*, but not for familiarity seekers.

**Figure 3:**
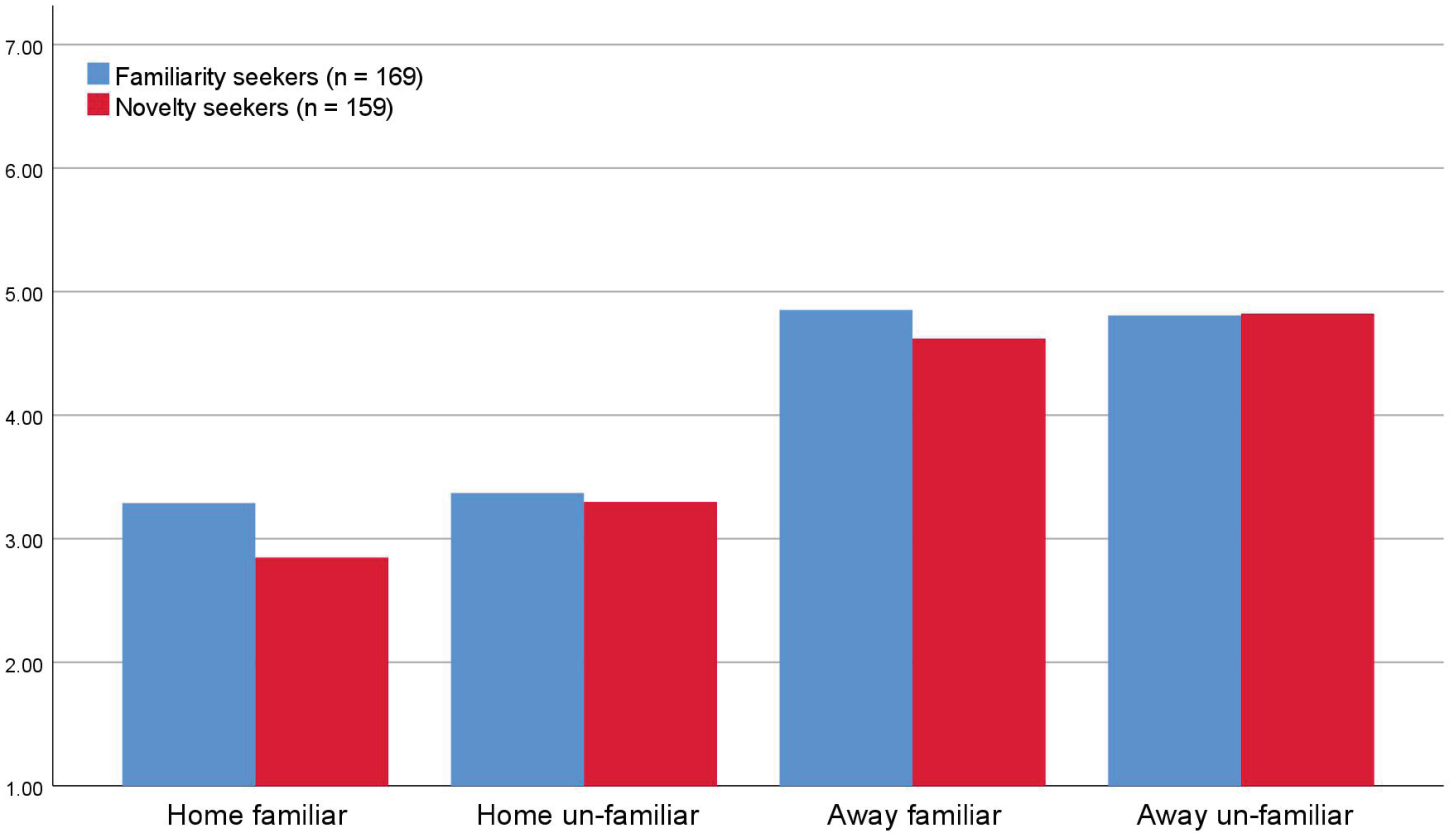
Novelty seekers and familiarity seekers preferences for combinations of novelty and familiarity [scale 1 (low interestingness) – 7 (high interestingness)].

Therefore, the second question in Study 2 was whether familiarity seekers and novelty seekers differ from each other in terms of what they judge to be interesting. Figure 3 shows that familiarity seekers and novelty seekers exhibit practically the same preference structure concerning the combination of novelty and familiarity. A one-way ANOVA, using novelty/familiarity seeking (the 25% of the respondents scoring the highest and the lowest on novelty seeking) as a grouping variable showed that familiarity seekers had significantly higher preference for familiar sights in familiar settings (home), but no other differences between the groups were observed concerning degree of interest in any of the scenarios. *Home/familiar*: mean_(familiarity seekers)_ = 3.36, mean_(novelty seekers)_ = 2.85, *F*_(1,325)_ = 6.26, *p* < 0.05; *home/novel*: mean_(familiarity seekers)_ = 3.36, mean_(novelty seekers)_ = 3.30, *F*_(1,324)_ = 0.11, *p* = 0.74; *away from home/familiar*: mean_(familiarity seekers)_ = 4.86, mean_(novelty seekers)_ = 4.62, *F*_(1,325)_ = 1.78, *p* = 0.18; *away from home/novel*: mean_(familiarity seekers)_ = 4.81, mean_(novelty seekers)_ = 4.81, *F*_(1,325)_ = 0.001, *p* = 0.98. In other words, novelty seekers and familiarity seekers express the same degree of interest for the various combinations of novelty and familiarity of sights in unfamiliar settings, but familiarity seekers have a higher preference for familiar sights in familiar places.

### Discussion

The first results from Study 2 are ambiguous in as much as respondents judge unfamiliar sights most interesting in both familiar and unfamiliar settings. Seemingly, people think of themselves that they to a large extent are novelty seekers, and that what attracts their interest is novelty more than familiarity. One reason for this may be that what attracts ones attention in familiar situations may be if there is something new in that situation, and that people, when they travel, tend to note what is different (novel) in the situation and not what is known. For example, if one is a first time visitor to a country and eats a salad (a well-known activity for most people), the ingredients or the toppings may be different from what one knows from home, and therefore people may notice this difference, not the fact that salad-eating is a well-known activity. This may lead people to conclude that they are attracted to, indeed find the novel taste the most interesting, which in turn may result in distorted self-perceptions of oneself as a person who not only likes, but is attracted to novelty (c.f., Teigen, 1987).

This interpretation is supported by the second finding in Study 2, which reveals that familiarity seekers and novelty seekers demonstrate similar preference structures concerning what constitutes an “interesting experience” – a result replicating the results in Study 1. Actually, this result indicates that tourists *think about themselves* that they are highly interested in novelty, not in familiarity. This represents a major methodological problem; how can the illusion that people apparently have of being predominantly interested in highly novel (exotic) sights and places be removed from the measuring of interestingness? Study 3 represents an attempt to extract this self-perception from peoples’ responses.

## Study 3: Interestingness for Typical Tourists

The results of Study 2 inspired a follow up study with the aim of focusing on what tourists think *other* tourists judge to be interesting. The study was a between subjects field experiment were tourists were randomized into either answering with reference to themselves as tourists or with reference to what they thought other tourists would find interesting. This was done for two reasons; first, it aimed at avoiding the confounding self-perception of being a person who is not a typical tourist (Prebensen et al., 2003; Doran et al., 2015, 2018), or indeed a better than average person (Alicke and Govorun, 2005; Brown, 2012). Second, the aim was to study and compare how the “self as tourist”-image and the perception of other tourists concerning our relevant parameters differed. It was expected that tourists would think that other tourists are more inclined to prefer familiar sights in novel settings than themselves. At the same time, it was expected that tourists would think of themselves as being significantly more interested in novel sights and experiences in unfamiliar settings than they would judge other tourists to be.

### Materials and Methods

Potential respondents were approached in “low threshold” places and asked if they were on vacation. If this initial question was answered in the affirmative, the tourists were asked if they would be willing to fill in a questionnaire concerning various “aspects of being a tourist.”

### Questionnaire

The questionnaires were four pages long, and took some 10 min to fill in. Trained research assistants distributed the questionnaires. Standard background questions, such as age, gender, and nationality, were asked, in addition to several items concerning various aspects of being a tourist. Respondents were randomized into four groups answering four versions of the questionnaire. The randomization procedure was that the questionnaires were distributed in a prefixed order securing that every participant had an equal probability of receiving any version of the questionnaire. Version 1 asked about the *attractiveness* of known and unknown sights in Norway and in the respondents home country; Version 2 asked about the *attractiveness* of named familiar (Edvard Grieg’s house) and unfamiliar (Amalie Skram’s house) sights in Bergen and (unnamed) familiar and unfamiliar sights in the respondents’ home town. In Version 3, respondents were asked to rate the *interestingness* of familiar and unfamiliar sights in Norway or in the respondents’ home country, and in Version 4, respondents were asked to rate the *interestingness* of named familiar and unfamiliar sights in Bergen and unnamed familiar and unfamiliar sights in their home town. Ratings were done on a 7-point scale anchored by “Not at all attractive” (or “Not at all interesting”) and “Very attractive” (or “Very interesting”).

About half of the respondents (*n* = 747) were asked to rate themselves as tourists with reference to novelty and familiarity of landmarks and sights. The first item was meant to tap interestingness of familiar sights and had this wording: “As a tourist, I visit famous landmarks instead of exploring unknown sights.” The second item aimed at extracting interestingness of unfamiliar (novel) sights and had the following wording: “As a tourist I visit unknown sights instead of exploring famous landmarks.” The remaining half (*n* = 751) of the respondents answered the same questions with reference to “typical first time tourists.” The wording in this version was “Tourists typically visit famous landmarks…” and “Tourists typically visit unknown sights…” Both groups indicated their response on a 7-point scale anchored by “Don’t agree at all” (1) and “Strongly agree” (7).

### Participants

Of some 1,650 approached tourists, 1,516 agreed to fill in the questionnaire. The respondents represented 43 nations, 51.6% were female and 48.4% male. Mean age was 47.7 years (SD = 17.91).

### Results

**Figure 4:**
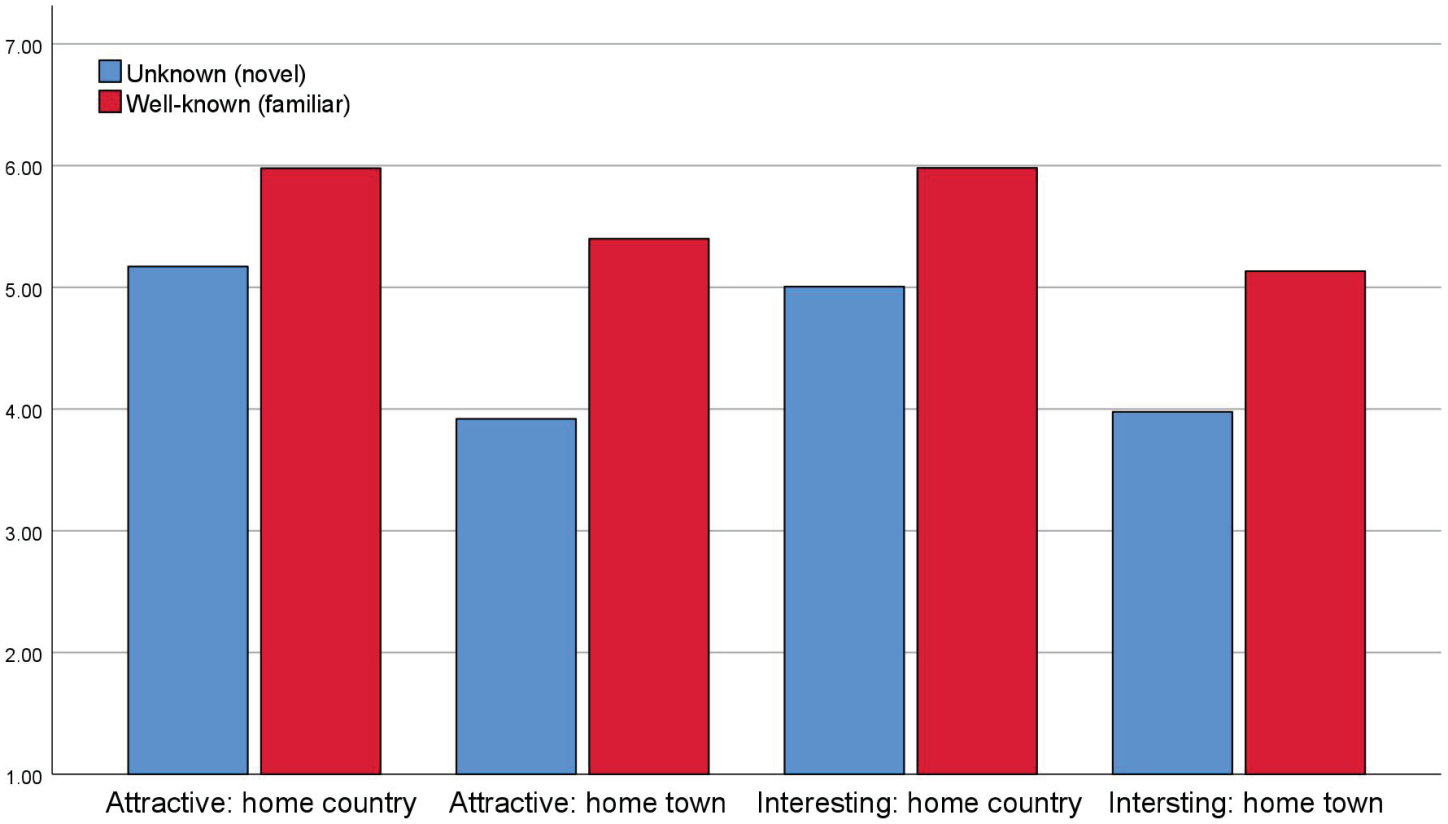
Attributed attractiveness and interestingness for novel and familiar attractions in home country and home town [scale 1 (low attractiveness) – 7 (high attractiveness)].

Figure 4 shows that respondents, when asked what they think typical first time visitors to their home country and home town find attractive or interesting, it is the country’s or home town’s most well-known attractions that are judged to be both attractive and interesting to a significantly higher degree than less well-known sights and attractions. Tourists seem to think that “typical first time tourists” visiting their own home towns and home countries are indeed interested in the familiar and famous and not in the less famous and novel sights.

When asked about tourists to Norway and Bergen, respectively, the same pattern appears as is evident from Figure 6. Tourists think that other “typical first time tourists” to Norway and Bergen are mostly attracted to well-known (familiar) sights and less to unknown (novel) sights. Similarly, the respondents stipulate that typical first time visitors find famous landmarks and sights more interesting both in Bergen and in Norway.

**Figure 5:**
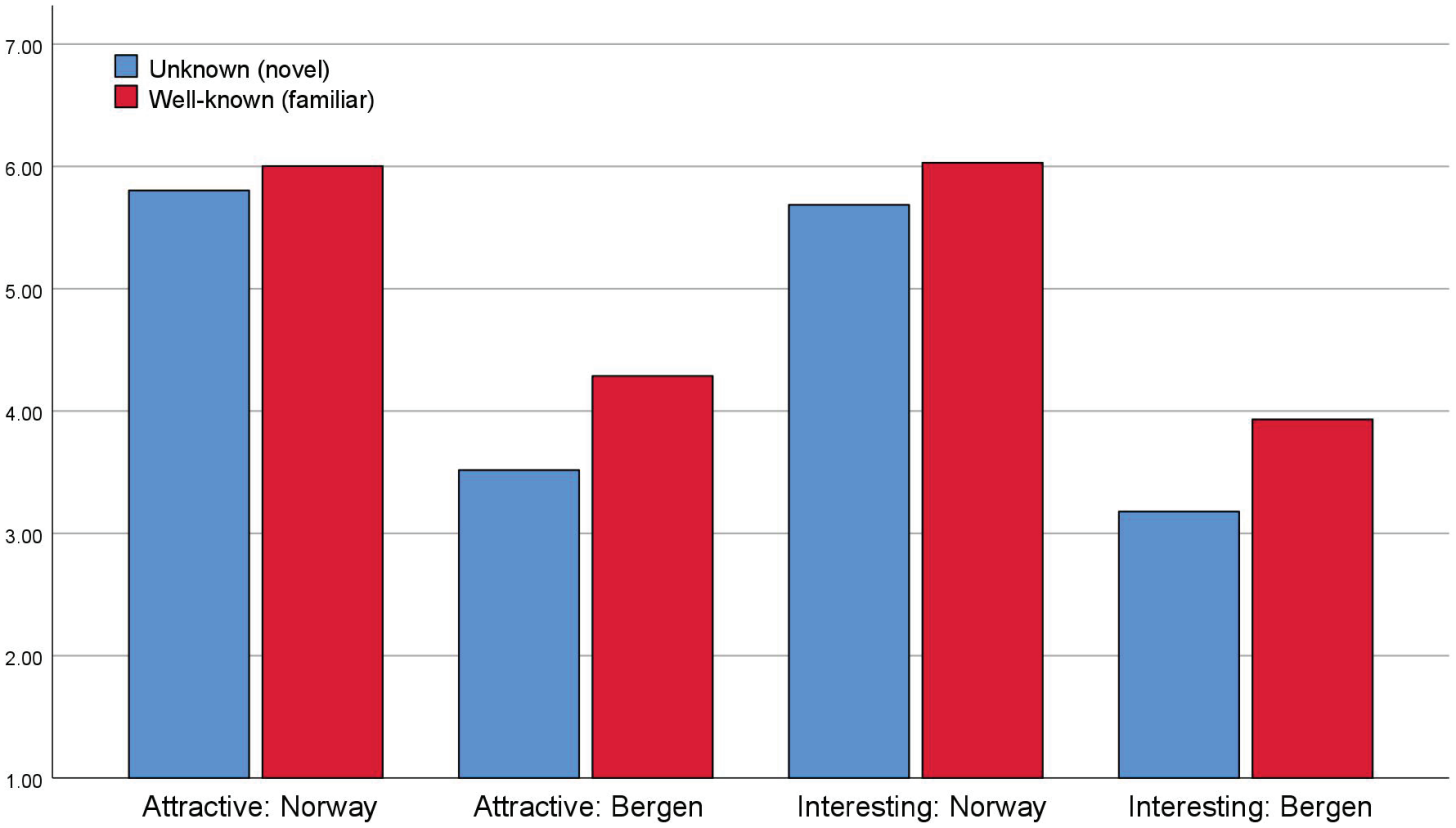
Attributed attractiveness and interestingness for novel and familiar attractions in Norway and Bergen [scale 1 (low attractiveness) – 7 (high attractiveness)].

It is probably noteworthy from Figures 4, 5 that “home town” (and “Bergen”) both seem to be less attractive and interesting than “home country” and “Norway” alike.

**Figure 6:**
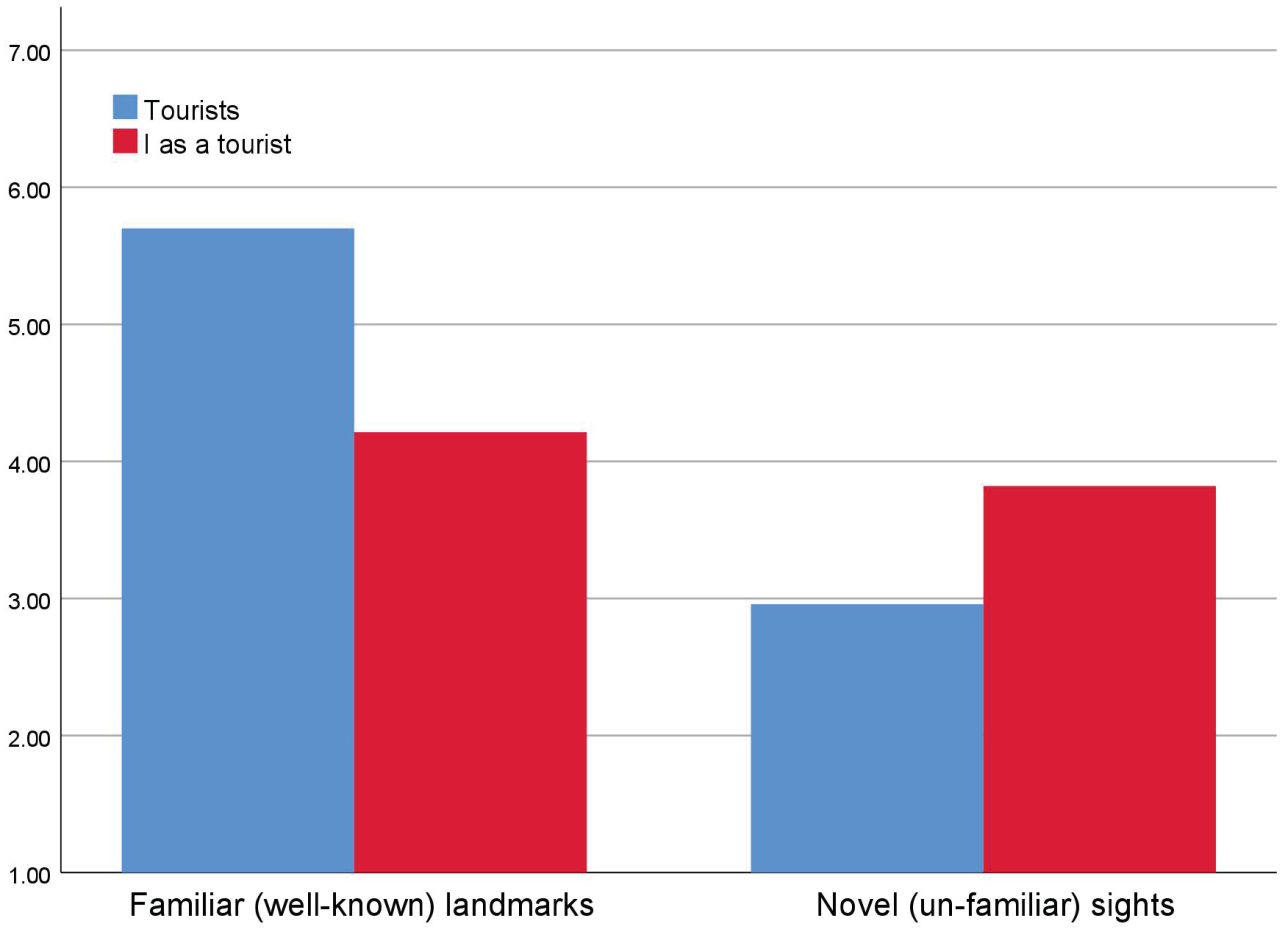
Interestingness of novel and familiar sights and attraction for “tourists” and for “me as a tourist” [scale 1 (low interestingness) – 7 (high interestingness)].

As can be seen from Figure 6, respondents think that “tourists” find familiar landmarks significantly more interesting than novel (unfamiliar) attractions (mean tourists_(familiar landmarks)_ = 5.70, mean tourists_(novel attractions)_ = 2.96, *t* = 35.02, *p* < 0.001). Also, as is evident from Figure 6, tourists report that they are themselves more interested in familiar than novel aspects of experiences (mean self_(novel attractions)_ = 3.82, mean self_(familiar attractions)_ = 4.21, *t* = 4.51, *p* < 0.001).

None the less, calculating the discrepancy score between expressed interestingness of familiarity and expressed interestingness of novelty yields an estimate of netto interestingness of novelty. Comparing the means of these discrepancy scores show that respondents to a significantly higher degree estimate that tourists are interested in familiarity than in novelty as compared to themselves in their roles as tourists (mean tourists_(net interestingness of familiarity tourists)_ = 2.74, mean self_(net interestingness familiarity)_ = 0.39, *F* = 403.08_(1,472)_, *p* < 0.001). This reflects that tourists think of themselves that they find novelty significantly more interesting while visiting places for the first time than what they judge other tourists to find. Respondents think that “typical first time tourists” find familiar sights to be significantly more interesting and novel sights to be significantly less interesting. One tends to think that tourists (not me) on their first time visit find it most interesting to look up well-known (familiar) sights, while the most uninteresting for such tourists are novel sights.

### Discussion

Results in Study 3 show that people think that typical first time visitors to unknown places will find well-known attractions to be most interesting and attractive. In addition, results indicate that people, when they are tourists, think that they are distinctly different from “typical first time visitors” in what they judge to be interesting. People think of other tourists that they look for well-known sights and hallmarks in new places, thus combining familiarity (well-known) and novelty (unknown) in construing the interestingness of a tourist experience. At the same time, people seem to think about themselves that they are significantly more balanced in what they would find interesting and attractive, and significantly less inclined “just to go for the tourist attractions.” This bears a resemblance to earlier findings indicating that people do not see themselves as “typical tourists” (Prebensen et al., 2003; Larsen and Brun, 2011; Doran and Larsen, 2014; Doran et al., 2015, 2018). Based on these results, one could actually suggest that this tendency is generic and applies over a range of tourist related behaviors, emotions, and cognitions. But most of all, results from Study 3 indicate that tourists think that other tourists look for familiarity while they think of themselves that they prefer an optimal blend of novelty and familiarity in their quest for interesting experiences.

## General Discussion and Conclusions

Study 1 revealed that tourists in general prefer to meet compatriots and *not* local people when they travel to countries that are unknown to them. At the same time, people prefer to meet *un*familiar people in more well-known settings. This was found to be true for novelty seekers and familiarity seekers alike. Study 2 revealed that tourists tend to think of themselves that they are novelty seekers and that they think of themselves that they prefer more exotic over less exotic experiences. Just like in Study 1, results from Study 2 indicated that the preference structures were similar in novelty seekers and in familiarity seekers alike. Results from Study 3 demonstrate that tourists think of other tourists that they are mostly interested in familiar sights in novel situations. Results from Study 3 also indicate that tourists think that other tourists are much more familiarity seeking than they are themselves in novel places.

The starting point of the present study was to test the predictions of traditional tourist role theory and those of the interaction hypothesis of inherent interest, as shown in Figure 1. It is evident from the results that the interaction hypothesis gets substantially more support than the tourist role perspective in the current material. This result is fascinating, since the cognitive model is virtually non-existent in the tourism literature, and at the same time, the tourist role orientation perspective has been highly influential in that same literature. This leads to a few thought-provoking preliminary conclusions that can be drawn on the basis of the joint findings of the three studies reported in the present paper.

The first conclusion is that the well-known platitude from general psychology, that people do not know what causes them to feel, think, and behave (Flanagan, 1991) seems to hold true also within the realm of inherent interest of tourist experiences. Seemingly, people think of themselves as not being familiarity seekers; they rather tend to think that they are novelty seekers. But, the results in the present series of studies also indicate that inherent interest in various situations seems to be a function of both familiarity and novelty just as predicted from the interaction hypothesis and documented in several earlier experiments from the psychology laboratory (Teigen, 1985a,b,c, 1987). One explanation why people think they are mostly interested in novelty may be that people overlook familiar aspects in situations containing something novel. This, in turn, may lead to screwed perceptions of oneself as a novelty seeker. In addition, this self-construal may lead to distorted memory processes; one will tend to remember the novel and not the mundane aspects of tourist experiences. It is well-known form the memory literature that for autobiographical memories, stimuli containing emotional arousal, i.e., novel stimuli (Talarico et al., 2004; Gilboa et al., 2018) are those that will be remembered most vividly. This implies that people do not know what makes things interesting for them and they do not know why particular experiences are more attractive than other experiences. Thinking that novel experiences are the most interesting ones may just be a memory distortion.

Another preliminary conclusion also emerging from the data is that although most tourists seem to think about themselves that they are novelty seekers, most people still prefer an optimal balance of novelty and familiarity in their tourist experiences. Actually, no differences were found between self-proclaimed novelty seekers and self-proclaimed familiarity seekers in terms of their preference structures: novelty seekers and familiarity seekers demonstrated parallel preference structures. This may imply that segmentation of people based on self-proclaimed preferences of novelty and familiarity may be futile. But even more importantly, these findings might also imply that classical tourist role theory is less feasible than thought by many tourism researchers. It is of course true that people travel for many reasons, but the idea that people travel to explore novel situations, unknown cultures, and unknown people do not seem to be completely true. Au contraire, based on the results from the present study, novelty seeking cannot be judged to be an exclusive and true motive for traveling neither for novelty seekers nor for familiarity seekers.

The third conclusion that may be drawn from the present study is that people, when traveling as tourists, tend not to think that they are like “other tourists.” This, of course is well-known from the literatures of both psychology and tourism research. Psychologists have, for example, amply documented the so-called “optimistic bias” (Weinstein, 1989) and the “better than average effect” (Alicke and Govorun, 2005; Brown, 2012) over a large range of domains, such as, for example, smoking (Arnett, 2000) and other health risks (Weinstein, 1987). It seems that people tend to think that they are unique in the sense that they are less likely to suffer negative outcomes and more likely to experience positive future outcomes, and that they fall prey to thinking that they perform better or have better abilities than average persons. Along similar ways of reasoning, Prebensen et al. (2003) reported that 89.5% of their sample of German tourists to Norway reported that they were not “typical German tourists”, while Larsen and Brun (2011) observed that tourists judged other tourists to be more at risk than themselves. Similarly, Doran et al. (2015) found that tourists asserted that they were not similar to other tourists in terms of their motivations, and Larsen et al. (2007) found that people tend to judge home country to be safer that abroad no matter what home country people come from. One possible interpretation of these findings is that people seem to find an optimal distance between their own self-perception and what they think other tourists represent in terms of many aspects of the tourist experience. It may well be that the search for familiarity is a sign of “typicality” in touristic terms, but it may equally well be that many, if not all tourists fall prey to the cognitive distortion of not being a typical novelty seeker. Seemingly, the pervasiveness of preference for familiarity in novel situations and novelty in familiar situations is a common characteristic of many, if not all tourists.

In sum, the present series of studies give stronger support for the cognitive interaction hypothesis of inherent interestingness then for the predictions of classical tourist role theory. The findings are in line with Teigen’s (1987) results. While Teigen based his conclusions on findings from laboratory settings among university students, the present studies were done in real life settings among tourists, which is a major advantage in terms of ecological validity. While findings from the psychology laboratory are of the greatest importance for the advancement of psychological science, such findings are always strengthened by corroborating findings from “real world” settings, such as in the present study. Those “institutionalized” and “noninstitutionalized” tourists do not differ in their preference structure for novelty and familiarity is compelling. Although “drifters” and “explorers” feel that they are distinctively different from other tourists in terms of novelty seeking, they are probably more similar to other tourists than they are aware of. Almost everyone tends to perceive themselves as different from the “mainstream tourist,” the “other tourists,” the “typical tourist” (c.f., Larsen et al., 2011) which in a sense makes them, or all of us, more typical than we think.

It should also be underlined that the results reported in the present study are not meant to give a complete and final solution to the question of what constitutes interesting tourist experiences. As Teigen (1987) highlighted, many psychological factors may play a role in peoples’ quests for such attractive experiences, such as, for example, emotional appeal, associative appeal, and personal appeal. Other sources of interestingness may be associated with feelings of well-being and peoples’ experiences of mastery. In addition, tourists’ worries and risk judgments may play a role. It must also be mentioned that it is not certain that this optimal blend of novelty and familiarity holds true for all classes of experiential domains; it may be that, for example, food and drink experiences and other experiences containing the possibility for disgust may turn out to be different. In addition, peoples’ expectancies to particular tourist places and events may also influence real life experience as exemplified in the well-known Jerusalem syndrome and in the less well-known Paris-syndrome (Flinn, 1962). However, it is our contention that only future research will contribute to dissolving the various highly interesting *general* psychological issues enclosed within the agenda of the study of “interestingness of tourist experiences.” Based on the findings reported in the present study, however, it is evident that the simplistic model which places people in categories based on their self-reported preferences for novelty and familiarity is not sufficient for enhancing the advancement of knowledge of the psychology of the tourist experience.

## Data Availability

Raw data supporting the conclusions of this manuscript will be made available by the authors, without undue reservation, to any qualified researcher.

## Ethics Statement

The data collection in the three studies in the present paper complied with the general guidelines for research ethics by the Norwegian National Committees for Research Ethics in the Social Sciences and the Humanities (NESH). Formal approval by an ethics committee was not required as per applicable institutional guidelines and regulations. Informed consent was implied by responding to the questionnaire in all three studies.

## Author Contributions

All authors contributed to the conception and design of the study. Data collection was carried out by student research assistants. SL and KW contributed to the statistical analysis, and SL wrote the first draft of the manuscript. All authors contributed to manuscript revision, read, and approved the submitted version.

### Conflict of Interest Statement

The authors declare that the research was conducted in the absence of any commercial or financial relationships that could be construed as a potential conflict of interest.
